# Everybody's Page

**Published:** 1891-06-20

**Authors:** 


### Everybody's Page.

According to Professor Forell, "Alcohol, together with the worship of the Golden Calf, is the true Devil of the nineteenth century."

Russian universities are more go-ahead than the Russian State. They intend, it is stated, establishing courses for the instruction of young women as druggists. There will be a new opening for the Nihilists.

The scheme for building artisans' dwellings on the site of Millbank Prison appears to have fallen to the ground, and from a sanitary point of view we think it is well it has. Millbank has always had an evil reputation, possibly arising from insanitary defects in the prison rather than in the actual site itself, and any risk of creating a centre of disease by the aggregation of numerous families on a malarious soil should be avoided at any cost.

Attention is called in the *Journal de Médecine de Paris* to a new hypnotic which certainly has one great merit, that of being quite harmless. Of its efficacy, not having tried it, we are unable to say anything. The mixture in question consists of equal parts of tartaric acid and bicarbonate of soda, the latter being dissolved in water, and the tartaric acid then added. Half to two drachms of the mixture is taken at bedtime, and should be drunk while effervescing.

A good deal has recently been written on "omnibuses," and the statement that few people have probably been inconvenienced by their absence during the strike has called forth many indignant protests. There is no doubt a certain amount of truth in the remark that the demand for this class of vehicle is immensely stimulated by its presence, but it is rather a stretch of imagination to say that under the influence of penny fares Londoners are rapidly forgetting how to walk. It is probably nearer the mark to say that many people to the detriment of their health are beguiled by the tempting wiles of the conductor to throw away opportunities for exercise which are the only daily ones they possess.

Soup does not sound an exhilarating drink for the breakfast table, yet Dr. Koch is credited by a German paper with preferring it to tea or coffee.

Unfortunate Ireland has another grievance. Hitherto the Emerald Isle has been credited with possessing a practical monopoly of ether drinking. The State of Michigan has cruelly robbed her of it, and the Finns, Swedes, and Poles there are said to drink ether with alcohol or whiskey on a large scale.

Essence of cinnamon is asserted to have been used with excellent results in the hospital in Marguelane, in Turkestan, in the treatment of all forms of malaria. Spraying the essence several times during the day in the wards is alleged to have proved more efficacious than eucalyptus, and to have met with success in cases which have resisted the action of quinine and arsenic.

The inhabitants of Liverpool are feeling uneasy as to the result of the census, and their uneasiness is only a reflection of an unpleasant feeling manifesting itself in other large centres of population. During the past ten years the returns of the medical officers have been prepared upon the basis that there would be a similar increase of population between 1881 and 1891 as between 1871 and 1881. It has already been shown that the death rates of our large towns have been calculated upon an unwarrantable and false assumption, and that the population of 21 towns has been over-estimated to the extent of 700,000! This means that the death rate upon which we have been congratulating ourselves has also been wrongly calculated, and that the deaths per thousand of the population have been considerably in excess of what they have been stated to be. This is a serious matter, and points to the probability, or rather fact, that we have been somewhat premature in glorifying ourselves upon the advance we have made in sanitary science.

